# Reference Interval Estimation from Mixed Distributions using Truncation Points and the Kolmogorov-Smirnov Distance (kosmic)

**DOI:** 10.1038/s41598-020-58749-2

**Published:** 2020-02-03

**Authors:** Jakob Zierk, Farhad Arzideh, Lorenz A. Kapsner, Hans-Ulrich Prokosch, Markus Metzler, Manfred Rauh

**Affiliations:** 1 Department of Pediatrics and Adolescent Medicine, University Hospital Erlangen, Erlangen, Germany; 2 Center of Medical Information and Communication Technology, University Hospital Erlangen, Erlangen, Germany; 3 Institute of Clinical Chemistry, University of Cologne, Cologne, Germany; 4 Chair of Medical Informatics, Friedrich-Alexander-University Erlangen-Nuremberg, Erlangen, Germany

**Keywords:** Diagnostic markers, Laboratory techniques and procedures, Diagnostic markers

## Abstract

Appropriate reference intervals are essential when using laboratory test results to guide medical decisions. Conventional approaches for the establishment of reference intervals rely on large samples from healthy and homogenous reference populations. However, this approach is associated with substantial financial and logistic challenges, subject to ethical restrictions in children, and limited in older individuals due to the high prevalence of chronic morbidities and medication. We implemented an indirect method for reference interval estimation, which uses mixed physiological and abnormal test results from clinical information systems, to overcome these restrictions. The algorithm minimizes the difference between an estimated parametrical distribution and a truncated part of the observed distribution, specifically, the Kolmogorov-Smirnov-distance between a hypothetical Gaussian distribution and the observed distribution of test results after Box-Cox-transformation. Simulations of common laboratory tests with increasing proportions of abnormal test results show reliable reference interval estimations even in challenging simulation scenarios, when <20% test results are abnormal. Additionally, reference intervals generated using samples from a university hospital’s laboratory information system, with a gradually increasing proportion of abnormal test results remained stable, even if samples from units with a substantial prevalence of pathologies were included. A high-performance open-source C++ implementation is available at https://gitlab.miracum.org/kosmic.

## Introduction

Laboratory test results need to be accompanied by appropriate reference intervals to support clinical decision-making^1,2^. Conventional protocols for the establishment of reference intervals (“direct methods”) require the sampling of a carefully selected, sufficiently large (usually >120 individuals), and homogenous group of healthy reference individuals, and the 2.5^th^ and 97.5^th^ percentiles of test results define the reference interval^3–5^. However, this approach is associated with substantial financial and logistic challenges, subject to considerable ethical restrictions in pediatrics, and limited in older age groups due to the high prevalence of chronic morbidities and medication^5,6^. Additionally, the use of direct methods results in substantial differences between the reference population and the target population in which the reference intervals are eventually used. Most patients are significantly older, use prescription drugs, and have relevant co-morbidities^1^. This results in uncertainty regarding the suitability of reference intervals based on blood samples from young and healthy adults, and limits the creation of accurate reference intervals for children and elderly adults. Importantly, many reference intervals for laboratory tests in children established using direct methods do not appropriately account for the extensive changes with age. Furthermore, use of conventional reference interval methods often results in inacceptable wide confidence intervals, particularly when non-normal distributions of test results are analyzed, e.g. analytes following a log-normal distribution^7^.

Indirect methods use data from laboratory information systems, which contain both physiological and abnormal test results, to overcome the restrictions mentioned above^7–9^. The basic assumption underlying these methods is that the majority of test results obtained during routine patient care are physiological, and can therefore be used to derive reference intervals^9^. To accomplish this, the proportion of physiological samples in the mixed input dataset is identified using different sophisticated statistical methods. As large numbers of test results are readily available from laboratory information systems, this enables the establishment of reference intervals specific to different populations, age-groups, analytical devices, and even batches and reagents. Extensive experience with these methods exists in children, where unique ethical challenges limit access to blood samples to create reference intervals^8,10–12^ and in challenging adult populations with a high proportion of patients with substantial morbidity and mortality^13^.

A variety of indirect methods have been implemented^5^, including the well-known Hoffmann approach^14^ and the Bhattacharya method^15^. However, both of these methods assume a Gaussian distribution of physiological test results, and require visual identification of a proportion of purely physiological test results, a process which is prone to bias and prevents integration into automated pipelines. Recently, a method developed by Arzideh *et al*.^9,16–19^ has been used to establish reference intervals for adults^13^ and children^8,10–12,20^. This method uses a truncation interval of the range of test results in the input dataset after Box-Cox transformation to estimate a distribution of supposedly physiological test results, and can therefore estimate non-Gaussian distributions. The truncation interval, the Box-Cox transformation parameter *λ*, and the parameters of the Gaussian distribution *µ* and *σ* are estimated using an elaborate statistical process, which is implemented within a freely available software package (https://www.dgkl.de/verbandsarbeit/arbeitsgruppen/entscheidungsgrenzen-richtwerte/). However, implementation using both Microsoft Excel and the R software environment requires human interaction and prevents integration into analysis pipelines, leads to technical difficulties, poor performance (reference interval estimation can take minutes), and the resulting lack of confidence intervals limits more widespread use and enhancement of this approach. Additionally, the statistical approach has evolved over time, and a complete and succinct description of the currently distributed algorithm has not been published, as has an in-depth evaluation of the method’s performance in terms of validity of the generated reference intervals.

As part of the PEDREF study (Next-Generation Pediatric Reference Intervals, www.pedref.org), in which pediatric reference intervals are established using data mining, we have created a high-performance implementation which uses an enhanced statistical approach. The developed application can be integrated into analysis pipelines and frameworks and provides confidence intervals for the estimated reference intervals. Here, we present the used algorithm, and evaluate the accuracy of the calculated reference intervals using both simulated datasets and patient samples. To facilitate evaluation of the algorithm, a web-based application allows analysis of datasets without local installation of the provided tools.

## Methods

We employ an approach based on previous works by Arzideh *et al*.^9,16–19^ and our experiences in their application to pediatric and adult datasets^8,10–13^: This procedure is based on the assumption that the proportion of physiological samples in the input dataset can be modeled with a parametric distribution (so-called Power Normal distribution, a Gaussian distribution after Box-Cox transformation of the data, i.e. a distribution that can accommodate skewed data), and that a truncation interval *T* exists within the dataset, in which the proportion of abnormal test results is negligible. Importantly, no assumptions regarding the distribution of pathologic samples are made.

**Figure 1:**
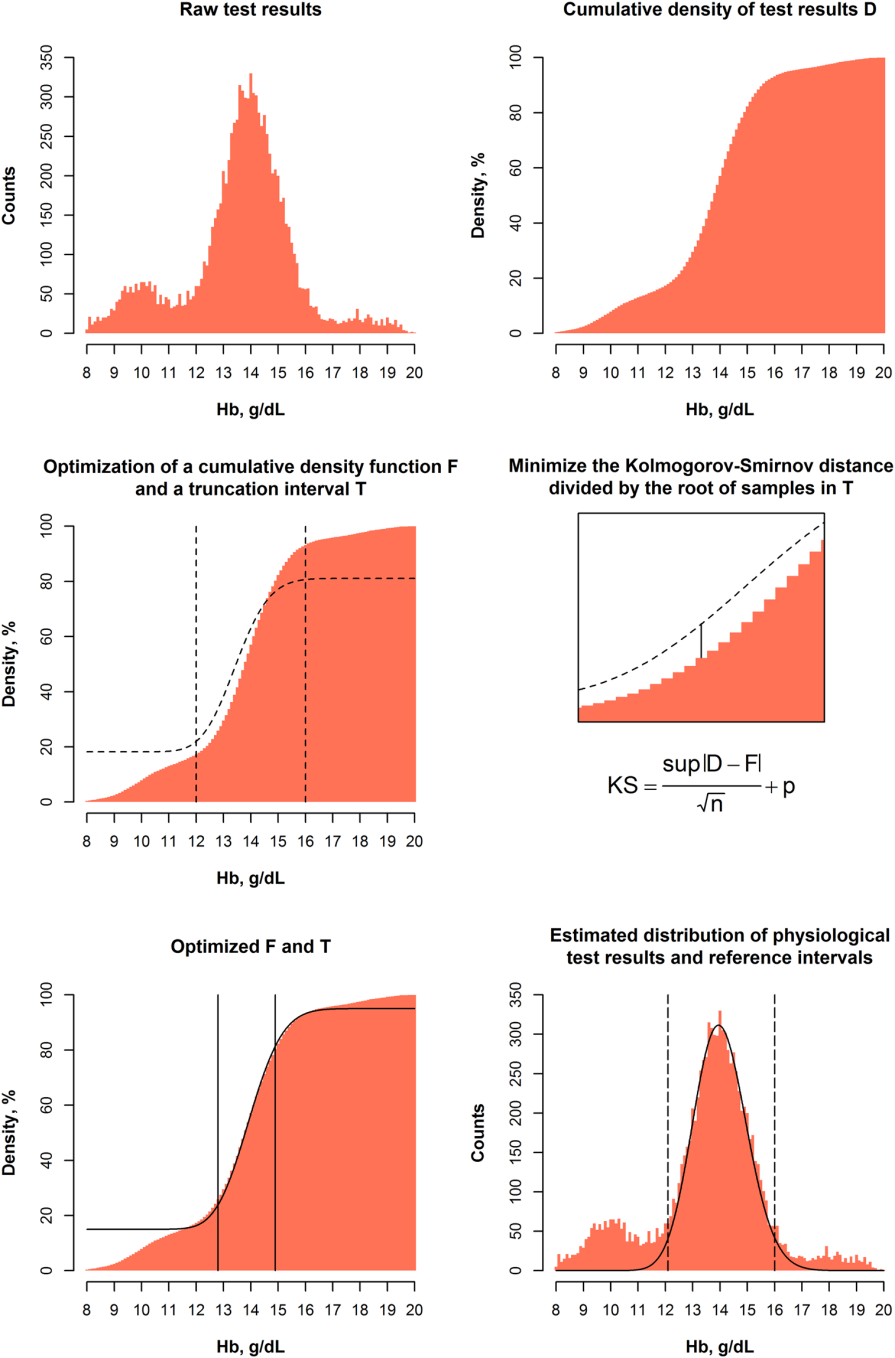
Estimation of reference intervals in a “contaminated” dataset. Based on the histogram of test results, the cumulative density (cumulative histogram) of test results *D* is calculated. After that, the cumulative density *F* of a parametrical function (a normal distribution described by *µ* and *σ*) is compared to *D* inside a truncation interval *T*, specifically, the maximum distance on the y-axis between *F* and *D*. Using an optimization process, *T* and the parameters *µ* and *σ* resulting in the minimum *sup |D-F|*/*√n + p* are identified (*n* denotes the number of samples inside the truncation interval, *p* denotes a penalty term, see Methods for details), which can be used to construct the estimated distribution of physiological test results. To enable the estimation of non-normal distributions, this process is performed for different “skewness” factors *λ*, which are applied beforehand (Box-Cox transformation using *λ*), and the *λ* resulting in the minimum optimization term *KS* is ultimately used.

The algorithm minimizes the Kolmogorov-Smirnov distance between an estimated normal distribution *F* and a truncated part of the observed distribution of test results after Box-Cox-transformation *D* (Fig. 1)^21,22^. This accounts for the fact that the majority of physiological biological distributions can be described using Gaussian distributions, Log-Normal distributions, or Gaussian distributions after Box-Cox transformation^23^, and that the Kolmogorov-Smirnov test is an established test for normality. The parameters of the normal distribution (*µ*, *σ*), the Box-Cox-transformation parameter (*λ*), and the truncation interval *T* are optimized numerically^24^. Specifically, the following term *KS* is minimized within the truncation interval *T*:

1 $$KS=\frac{\sup |D-F|}{\sqrt{n}}+p$$

where *D* denotes the cumulative density function of the dataset after Box-Cox transformation using *λ*, *F* denotes the cumulative density function of a normal distribution described by *µ* and *σ*, and *n* denotes the number of samples within *T*. *p* denotes a penalty term for test results outside the truncation interval, defined as the sum of *p*_1_ and *p*_2_

2 $${p}_{1}=\frac{\sup \,F-D}{\sqrt{n}}$$

3 $${p}_{2}=\frac{\sup \,D-F}{\sqrt{n}}$$

outside the truncation interval *T* (*p*_1_ is calculated for values below T, *p*_2_ is calculated for values greater *T*, *p*_1_ or *p*_2_ are ignored if either is <0).

In practice, the following steps are performed in a nested manner (Fig. 1):

1. Optimization of *λ* (this is done using iterative “Brute Force search” optimization, i.e. every *λ* in 0, 0.1, 0.2, …, 1.0 is tried, followed by *λ* − 0.09, *λ* − 0.08, …, *λ* + 0.09, restricted to [0.0 to 1.0]).
2. Optimization of *µ* and *σ* using Downhill-Simplex/Nelder-Mead optimization.
3. Optimization of the truncation interval *T* (“Brute Force search” optimization, i.e. every possible combination of the lower truncation limit *T*_1_ and upper truncation limit *T*_2_ [within bounds specified as input parameters, by default the 5^th^ to 30^th^ percentile, and 70^th^ to 95^th^ percentile of the input dataset, respectively] is examined).
4. Calculation of the optimization target (minimization target) *KS*.

We selected “Brute Force search” optimization for steps 1 and 3, as unpredictable local minima prevent the use of other minimization techniques. To provide confidence intervals, we use bootstrapping of the input dataset (random sampling with replacement). A high-performance open source C++-implementation of the outlined algorithm is available as part of the *PEDREF* study (Next-Generation Pediatric Reference Intervals, Kolmogorov-Smirnov based reference intervals, *kosmic* at https://gitlab.miracum.org/kosmic). The application is small (<1 MB binary), portable (compiles under Windows and Linux without external dependencies) and uses multi-threading for optimum performance on modern computers. Python bindings (Python Software Foundation, https://www.python.org/) enable integration of *kosmic* as a binary library into custom analysis pipelines, and a web-based tool available at https://kosmic.diz.uk-erlangen.de/ enables use of the presented application without local installation.

To evaluate the algorithm’s performance in terms of the correctness of the generated reference intervals, we assessed the impact of abnormal samples on reference interval estimations using simulated datasets (“hemoglobin”, “Thyroid-stimulating hormone, TSH”, and “Gamma-glutamyltransferase, γ-GT”). We generated random distributions of physiological test results corresponding to typical adults’ reference intervals (“hemoglobin”: Gaussian distribution, μ_physiological_ = 14.0 and *σ*_physiological_ = 0.98 corresponding to a reference intervals from 12.0–16.0 “g/dL”; “TSH”: Log-Normal distribution, reference interval 0.25-4.0 “U/l”; “γ-GT”: Log-Normal distribution, reference interval 10–50 “U/l”). Sets of “abnormal” samples were added (“hemoglobin”: Gaussian distribution, varying μ_abnormal_ and *σ*_abnormal_; “TSH”: varying Gaussian distributions; “γ-GT”: varying Log-Normal distributions) while the total number of samples in the dataset was not varied (“hemoglobin”: n_total_ = 10,000; “TSH”: n_total_ = 50,000; “y-GT”: n_total_ = 25,000): We changed the ratio of “abnormal” samples (0%–30%) and the position of the distribution of “abnormal” samples. These steps were performed for n=100 cycles, and median estimated upper and lower reference limits are reported, as are the 90% confidence intervals of estimated upper and lower reference limits.

Similarly, we examined the influence of abnormal test results on reference intervals in a patient dataset from a tertiary care center (University Hospital Erlangen, Germany): We retrieved laboratory test results from patients aged 18–60 years from the laboratory information system (inpatients and outpatients from all units, including test results from specialty units and intensive care units). (Analysis of test results performed during patient care for research is in accordance with the applicable German/Bavarian regulations and does not require patients’ explicit consent. Use of pediatric and adult patient datasets in the PEDREF study has been approved by the Ethical Review Boards of the University Hospital Erlangen, reference number 97_17 Bc.) Test results were then categorized in groups according to the prevalence of abnormal samples in the requesting unit (A, B, C, D, with an increasing proportion of abnormal test results, see Table 1), and these groups were combined for further analyses (e.g. group AB contains test results from groups A and B). Only one randomly selected sample per patient was examined when estimating reference intervals, based on previous examinations, which showed no difference between a random sample and more selective sample selection approaches^13^. The patient population in the examined dataset consists mainly of Caucasian individuals and we did not stratify according to ethnicity.

**Table 1:** Patient samples categorized according to the estimated probability of blood count abnormalities.

Group	Estimated Probability of blood count abnormalities	Patient group	Samples	Patients
A	Very low, comparable to the general population	Hospital staff occupational health check-ups	15503	15493
B	Low	Endocrinology, Cardiology, Nuclear medicine	11872	5894
C	Intermediate	Remaining units, mainly internal medicine, including ERs	89171	20427
D	Very high	Oncology/hematology, radiation therapy, ICUs	81649	5932

Samples and patients columns show the median number of samples and patients per analyte in each group used for reference interval estimation (18–60 years). ER, emergency room; ICU, intensive care unit.

## Results

We provide a high-performance and open source implementation of an indirect method for reference interval estimation (https://gitlab.miracum.org/kosmic). Reference intervals can be calculated quickly, e.g. <50 ms using a typical dataset of hemoglobin test results without confidence intervals, and <3 s with confidence intervals using bootstrapping (n=100) on a typical personal computer. Importantly, the tool can be integrated into custom pipelines using a command line interface or using Python bindings on different computing platforms. For evaluation purposes, we provide a web-based application (https://kosmic.diz.uk-erlangen.de/), which enables use of *kosmic* without local installation.

**Figure 2:**
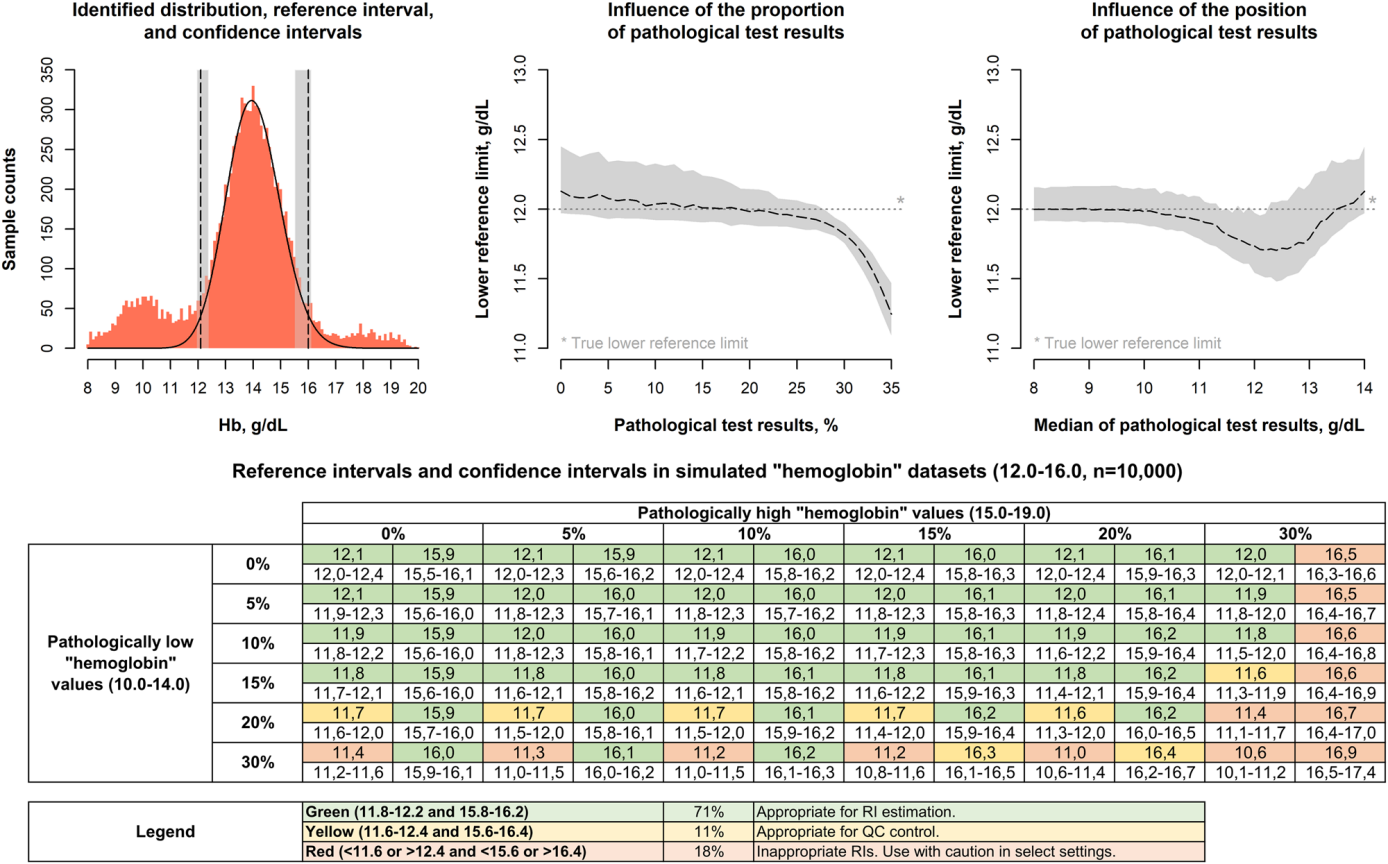
Influence of abnormal test results on estimated “hemoglobin” reference intervals. Reference intervals (dashed lines) and 90% confidence intervals (gray areas) for simulated “hemoglobin” datasets (true reference interval 12.0–16.0 “g/dL”) with different proportions of abnormal “hemoglobin” test results. The center-top panel shows the influence of the proportion of abnormal test results (95% interval of abnormal “hemoglobin” values 8.0–12.0 “g/dL”, n_total_ = 10,000) on estimated lower reference limits, the center-right panel shows the influence of the position of abnormal test results (20% abnormal “hemoglobin” values, 95% range 4.0 “g/dL”, n_total_ = 10,000). The bottom table shows “hemoglobin” reference intervals and confidence intervals when both abnormally high and low values are contained in the dataset (n_total_ = 10,000). (Supplemental Table 1 shows combinations of other abnormal “hemoglobin” distributions, and Supplemental Tables 2 and 3 show reference intervals for simulated “Thyroid-stimulating hormone, TSH”, and “Gamma-glutamyltransferase, γ-GT” test results).

Evaluations of the provided tool using simulated scenarios of common biological analytes with increasing proportions of abnormal test results and varying overlap of physiological and abnormal distributions are shown in Fig. 2 and Supplemental Tables 1–3. Simulated “hemoglobin” reference intervals (Fig. 2, Supplemental Table 1) show the relationship of generated reference intervals, position of abnormal test results, and ratio of abnormal and physiological test results, and the width of reference intervals’ confidence intervals. Importantly, most estimated reference limits (74%, Supplemental Table 1) are within narrow limits (11.8-12.2 and 15.8-16.2 “g/dL”) of the true reference limits (12.0 and 16.0 “g/dL”). In scenarios with the most overlap between physiological and abnormal test results (i.e. when the abnormal distributions are “centered” on the true lower or upper reference limit), estimations are most challenging, resulting in an increasing deviation of estimated and true reference limits, especially when the proportion of abnormal values is ≥20%. On the other hand, reference limit estimations are reliable even in challenging settings (up to 30% abnormal test results on either side), when the overlap between abnormal and physiological test results is low. When assessing the differences between estimated and true reference limits separately for lower and upper reference limits, differences in estimated and true reference intervals are more pronounced in upper reference limits.

Simulated scenarios of analytes that can be described using a Log-Normal distributions are shown in Supplemental Tables 2 and 3. Results in these simulations are in line with the “hemoglobin” results, specifically, a high proportion of reference limits within narrow limits (81.1% of “TSH” reference limits within 0.2–0.3 and 3.8–4.2 “mU/L”, and 65.9% of “γ-GT” reference limits within 8–12 and 48–52 “IU/L”), with more differences between true reference limits and estimated limits for the upper than the lower reference limit. Similarly, most estimated reference limits are within the specified margins when <20% of samples are abnormal, depending on the overlap between physiological and abnormal samples. Interestingly, upper reference limits for “γ-GT” are more often estimated too low rather than too high in comparison to “hemoglobin” and “TSH” reference intervals. (The abnormal samples for “γ-GT” are simulated using a Log-Normal distribution, while a Gaussian distribution was used for “hemoglobin” and “TSH”).

**Figure 3:**
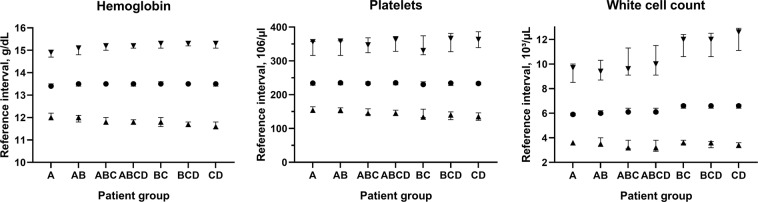
Estimated reference limits in different analytes and patient groups. Reference intervals and confidence intervals for hemoglobin concentration, white cell count, and platelet count in women were determined using different patient groups with an increasing proportion of pathologic samples (e.g. Group A: check-up visits only, Group D: hematology/oncology patients only, see Table 1 for full group descriptions). ▲ denote the 2.5^th^ percentiles, ■ denote the 50^th^ percentiles, and ▼ denote the 97.5^th^ percentiles, bars denote the respective 90% confidence intervals.

Reference intervals for 3 different common laboratory tests (hemoglobin, white cell count, and platelets) and associated 90% confidence intervals generated using samples from a tertiary care center’s laboratory information system are shown in Fig. 3. These results show a minor widening in reference intervals with an increasing proportion of abnormal test results, most pronounced in white cell count upper reference limits and least pronounced in platelet upper reference limits. Importantly, changes in reference intervals between groups A, AB, ABC, and ABCD are minor, although a substantial proportion of abnormal samples is included in datasets ABC and ABCD, specifically patients from emergency rooms, oncology, and intensive care units – in these patients a substantial prevalence of anemia (low hemoglobin concentrations), leukocytosis (high white cell counts) and leukopenia (low white cell counts), and thrombocytopenia (low platelet counts) has to be considered. Even when removing patient samples with a relatively low proportion of abnormal samples (i.e. groups A and/or B), reference intervals for hemoglobin and platelets remain relatively stable, whereas white cell counts upper reference limits show substantial changes in these settings.

## Discussion

For clinical laboratories, establishment and validation of reference intervals are essential tasks and partition of reference intervals by covariates, most importantly age and sex, is of major clinical importance. Direct approaches to reference interval calculation require recruitment of adequately sized healthy cohorts which account for all relevant covariates – a challenge which is often unmet, especially when individual laboratories establish reference intervals. Indirect approaches use samples from patient care for reference interval estimation, and are therefore unrestricted by ethical, practical, and financial limitations due to the need for sample acquisition.

We provide a high-performance and open source implementation of an indirect method for reference interval estimation. In contrast to previous implementations, which used both Microsoft Excel and R and therefore required human interaction, this enables integration into analysis frameworks. Additionally, we opted to provide *kosmic* under an open-source license (“GNU General Public License, Version 3.0”), which facilitates peer review of the implementation and enhancement of our approach – a process we greatly welcome. Implementation using C++ enables creation of dependency-free executables for different computing platforms, which simplifies distribution and mitigates technical difficulties. Device manufacturers can integrate *kosmic* into laboratory information systems and laboratory analyzers, to provide indirect methods for reference interval calculation as a complement to direct approaches. More importantly, the increased run-time performance enables the calculation of confidence intervals using bootstrapping.

Stratification of reference intervals by clinically relevant covariates requires a quantitative measure of reference interval precision, e.g. confidence intervals. Availability of confidence intervals for reference intervals in the provided implementation is therefore a marked improvement to previous implementations, and enables application of this indirect method to a broader field of clinical and scientific areas and questions. The possibility to integrate the algorithm into analytical frameworks is of major importance for comprehensive studies. In the PEDREF study, pediatric reference intervals are represented using continuous reference intervals and percentile charts, resulting in a separate reference interval for each day of life. To this end, hundreds of discrete reference intervals are estimated and joined using spline curves, an approach which requires embedding of the statistical algorithm. This is greatly facilitated by the availability of *kosmic* as a non-dependency executable and as a Python library. Importantly, this also simplifies replication and validation of results between different studies.

The reference intervals established with *kosmic* are reliable even in challenging simulation settings (i.e. when the distributions of abnormal test results are centered on the 2.5^th^ and 97.5^th^ percentiles of the distribution of physiological test results) when <20% test results are abnormal (Fig. 2, Supplemental Tables 1–3). When using patient samples, estimated hemoglobin, platelet count, and white cell count reference intervals were stable, even if patients from intensive care units and hematology/oncology are included (Fig. 3). Hemoglobin and platelet count reference intervals remained stable, even if samples from units with a low proportion of abnormal test results were removed and only samples from units with a substantial prevalence of pathologies remained (e.g. emergency and intensive care, hematology/oncology), while estimation of appropriate white cell count reference intervals depended on the presence of a relatively healthy patient group. The latter finding is probably due to the unspecific nature of leukocytosis, which can be caused e.g. by infection, inflammation due to other causes, and malignant diseases, but also by stress – a very common finding in a hospital population. (One could possibly argue that mild leukocytosis <13,000/*µl* is so unspecific in a hospital setting, that it can be considered essentially non-diagnostic – or “normal”.) Overall, these results confirm the basic assumption of indirect methods (correct identification of the proportion of physiological test results in a mixed dataset is possible), and the suitability of the statistical approach used to this end in *kosmic* (optimization of truncation points and a Gaussian distribution after Box-Cox-transformation using the Kolmogorov-Smirnoff distance) in a wide range of clinical scenarios.

Both simulated datasets and patient datasets show a more pronounced volatility of upper reference limits in comparison to lower limits. This is mainly caused by the assumptions underlying the statistical algorithm: the Box-Cox transformation parameter *λ* is optimized in the range [0, 1], resulting in distributions ranging from a symmetrical Gaussian (*λ* = 1) to a right-skewed Log-Normal (*λ* = 0) distribution. Depending on the input dataset, the algorithm therefore models the distribution of supposedly physiological test results with a right-skewed Log-Normal distribution, while a left-skewed distribution would be outside the specified parameter bounds. A mixture of two overlapping Gaussian distributions, with a major physiological part on the “left” and a minor abnormal part on the “right” results in a “Log-Normal-like” distribution, explaining the more pronounced difficulty of the algorithm in separating abnormally high and physiological test results in comparison to abnormally low test results.

The presented evaluations of *kosmic* using simulations and real-world datasets enable assessment of the algorithm’s applicability for different clinical and scientific scenarios, in contrast to other indirect approaches for reference interval estimation, where the performance in terms of reference interval accuracy has been less intensively studied. Importantly, this can be used to guide preprocessing of the input dataset, e.g. using filters (“remove patients from intensive care units”, “remove all patients with multiple hospitalizations”, “remove all patients with repeat measurements”) to reduce the proportion of patients with a high proportion of abnormal test results. Based on the results from our simulations and patient datasets, we recommend a proportion of less than 20% abnormal test results. Our results in patient datasets demonstrate that this does not necessarily require removal of patients even with a very high proportion of abnormal test results (e.g. patients from intensive care units). However, if removal of samples using meta-information e.g. regarding intensive care treatment or clinical information is feasible, this can certainly improve the accuracy of reference intervals.

## Limitations

*kosmic* can be used to establish reference intervals for homogenous populations. While this is an important clinical and scientific application, e.g. to create sex-specific reference intervals for homogeneous age groups (i.e. stratification using categorical features), many covariates change continuously. Specifically, we have shown continuous change with age of reference intervals in children and argued for a corresponding representation^8,10–12^ – however, establishment of continuous reference intervals, even with *kosmic*, currently requires a two-step process, in which reference intervals are first established for discrete age groups and then fused to create a continuous representation. We are currently exploring integration of nominal covariates into the algorithm, and availability of *kosmic* as open source software also enables other groups to improve our approach with regard to covariates or other features. Additionally, while we have greatly improved the run-time performance of the algorithm in comparison to previous implementations, some dataset characteristics can negatively impact run time. In particular, the time needed to identify the best truncation interval using a “Brute Force” approach depends on the number of possible combinations of upper and lower truncation points in the truncation interval search area (i.e. 5^th^ to 30^th^ and 70^th^ to 95^th^ percentile by default) – under unfavorable conditions (depending on the number of decimal digits and the shape of the input distribution), this can still result in long run times.

## Conclusions

*kosmic* enables accurate reference interval estimation using patient samples retrieved from laboratory information systems. This facilitates more widespread application of indirect approaches for reference interval calculation, with the ultimate aim to increase the value of laboratory testing for clinical decision-making.

## Supplementary information

Supplemental Tables.

## Data Availability

An open-source (GPL 3) C++ implementation and windows builds of the presented algorithm are available at https://gitlab.miracum.org/kosmic, the simulation datasets are available at https://gitlab.miracum.org/kosmic/benchmarks. A web-based application for evaluation purposes is available at https://kosmic.diz.uk-erlangen.de/. The patient datasets analyzed in the present report (Fig. 3) were used with permission from Prof. S.W. Krause (Department of Medicine 5 - Haematology and Oncology, University Hospital Erlangen, Erlangen, Germany) and are not publicly available. Data are however available from the authors upon reasonable request and with permission from Prof. S.W. Krause.
