# Supplementary material for: Reference Interval Estimation from Mixed Distributions using Truncation Points and the Kolmogorov-Smirnov Distance (kosmic)
Source: Sci Rep. 2020 Feb 3;10:1704. doi: 10.1038/s41598-020-58749-2 (PMC6997422; doi:10.1038/s41598-020-58749-2)
Supplement: Supplementary file 1 — Supplemental Tables. [file 41598_2020_58749_MOESM1_ESM.pdf]

Supplemental Material

Jakob Zierk, Farhad Arzideh, Lorenz A. Kapsner, Hans-Ulrich Prokosch, Markus Metzler, and Manfred Rauh:

Reference Interval Estimation from Mixed Distributions using Truncation Points and the Kolmogorov-Smirnov Distance (kosmic)

***Hemoglobin, 12-16 g/dl*, n=10,000**

Abnormal,%		None		13.0-17.0										14.0-18.0									
		0%		5%		10%		15%		20%		30%		5%		10%		15%		20%		30%	
None	0%	12,1 (12,0-12,5)	15,9 (15,5-16,1)	12,1 (12,0-12,4)	16,0 (15,7-16,2)	12,1 (12,0-12,4)	16,1 (15,8-16,2)	12,1 (12,0-12,4)	16,2 (15,8-16,3)	12,2 (12,0-12,5)	16,2 (15,9-16,4)	12,2 (12,0-12,5)	16,4 (16,0-16,5)	12,1 (12,0-12,4)	16,0 (15,7-16,2)	12,1 (12,0-12,4)	16,1 (15,7-16,4)	12,1 (12,0-12,4)	16,2 (15,9-16,5)	12,1 (12,0-12,4)	16,4 (16,1-16,7)	12,1 (12,0-12,2)	16,9 (16,5-17,1)
11.0-15.0	5%	12,0 (11,9-12,4)	15,9 (15,6-16,0)	12,0 (11,9-12,3)	16,0 (15,6-16,1)	12,0 (11,9-12,4)	16,1 (15,7-16,2)	12,1 (11,9-12,3)	16,2 (15,8-16,3)	12,1 (11,9-12,4)	16,2 (15,8-16,4)	12,1 (11,9-12,4)	16,4 (16,1-16,6)	12,0 (11,9-12,3)	16,0 (15,6-16,2)	12,0 (11,9-12,3)	16,1 (15,7-16,4)	12,0 (11,9-12,3)	16,2 (15,8-16,5)	12,0 (11,9-12,3)	16,3 (16,1-16,7)	12,0 (11,9-12,1)	16,9 (16,6-17,2)
	10%	11,9 (11,8-12,3)	15,9 (15,5-16,0)	11,9 (11,8-12,3)	16,0 (15,7-16,1)	12,0 (11,8-12,3)	16,1 (15,8-16,2)	12,0 (11,8-12,3)	16,2 (15,7-16,3)	12,0 (11,8-12,3)	16,2 (15,8-16,4)	12,0 (11,9-12,3)	16,4 (16,1-16,6)	11,9 (11,8-12,3)	16,0 (15,7-16,2)	11,9 (11,8-12,2)	16,1 (15,8-16,4)	11,9 (11,8-12,2)	16,2 (15,9-16,6)	11,9 (11,8-12,2)	16,4 (16,0-16,7)	11,8 (11,7-12,0)	16,9 (16,5-17,3)
	15%	11,9 (11,7-12,3)	15,9 (15,5-16,0)	11,9 (11,7-12,2)	16,0 (15,7-16,1)	11,9 (11,7-12,3)	16,1 (15,7-16,2)	11,9 (11,7-12,3)	16,2 (15,8-16,3)	11,9 (11,7-12,2)	16,3 (15,9-16,4)	11,9 (11,7-12,3)	16,5 (16,0-16,6)	11,9 (11,7-12,2)	16,0 (15,8-16,2)	11,9 (11,7-12,3)	16,1 (15,7-16,4)	11,8 (11,7-12,1)	16,2 (15,9-16,6)	11,8 (11,7-12,1)	16,4 (16,0-16,8)	11,7 (11,6-11,9)	16,9 (16,5-17,2)
	20%	11,8 (11,6-12,2)	15,9 (15,5-16,0)	11,8 (11,6-12,2)	16,0 (15,6-16,1)	11,8 (11,6-12,2)	16,1 (15,7-16,2)	11,8 (11,6-12,1)	16,2 (15,8-16,3)	11,8 (11,6-12,2)	16,3 (15,8-16,4)	11,8 (11,6-12,1)	16,5 (16,2-16,6)	11,8 (11,6-12,2)	16,0 (15,6-16,2)	11,8 (11,6-12,2)	16,1 (15,7-16,4)	11,7 (11,6-12,1)	16,2 (15,8-16,6)	11,7 (11,6-12,0)	16,4 (16,0-16,7)	11,6 (11,5-11,7)	17,0 (16,5-17,3)
	30%	11,6 (11,5-12,0)	15,8 (15,4-15,9)	11,6 (11,5-12,1)	15,9 (15,6-16,1)	11,6 (11,4-12,1)	16,1 (15,6-16,2)	11,6 (11,4-12,0)	16,2 (15,6-16,3)	11,5 (11,4-11,9)	16,3 (16,0-16,5)	11,5 (11,4-11,9)	16,5 (16,2-16,7)	11,6 (11,4-12,0)	15,9 (15,5-16,1)	11,6 (11,4-12,0)	16,1 (15,6-16,4)	11,5 (11,4-11,8)	16,2 (15,9-16,6)	11,5 (11,4-11,8)	16,4 (16,1-16,7)	11,4 (11,3-11,6)	16,9 (16,6-17,2)
10.0-14.0	5%	12,0 (11,9-12,3)	15,9 (15,6-16,0)	12,0 (11,8-12,4)	16,0 (15,6-16,1)	12,0 (11,9-12,3)	16,1 (15,7-16,2)	12,1 (11,9-12,4)	16,2 (15,8-16,3)	12,1 (11,9-12,4)	16,3 (15,9-16,4)	12,1 (11,9-12,5)	16,4 (16,1-16,6)	12,0 (11,8-12,3)	16,0 (15,6-16,2)	12,0 (11,8-12,3)	16,1 (15,8-16,4)	12,0 (11,8-12,3)	16,3 (15,9-16,5)	12,0 (11,8-12,3)	16,4 (16,0-16,8)	11,9 (11,8-12,1)	16,9 (16,6-17,3)
	10%	12,0 (11,8-12,2)	15,9 (15,6-16,0)	12,0 (11,8-12,3)	16,0 (15,6-16,1)	12,0 (11,8-12,3)	16,1 (15,8-16,2)	12,0 (11,8-12,3)	16,2 (15,8-16,3)	12,0 (11,8-12,3)	16,3 (16,0-16,4)	12,1 (11,8-12,4)	16,5 (16,1-16,6)	12,0 (11,8-12,3)	16,0 (15,7-16,2)	12,0 (11,7-12,2)	16,1 (15,9-16,4)	11,9 (11,7-12,2)	16,3 (16,0-16,7)	11,8 (11,6-12,3)	16,4 (16,1-16,9)	11,7 (11,5-12,1)	17,0 (16,6-17,6)
	15%	11,8 (11,7-12,2)	15,9 (15,6-16,0)	11,9 (11,7-12,1)	16,1 (15,9-16,1)	11,9 (11,7-12,2)	16,1 (15,8-16,2)	11,9 (11,7-12,2)	16,2 (15,9-16,4)	11,9 (11,7-12,3)	16,3 (16,0-16,5)	11,9 (11,8-12,2)	16,5 (16,2-16,6)	11,8 (11,6-12,1)	16,1 (15,8-16,2)	11,8 (11,6-12,2)	16,2 (15,9-16,4)	11,8 (11,5-12,2)	16,4 (16,0-16,7)	11,7 (11,4-12,1)	16,6 (16,2-17,0)	11,6 (11,2-12,0)	17,2 (16,7-17,8)
	20%	11,7 (11,5-12,0)	15,9 (15,7-16,0)	11,7 (11,5-12,0)	16,0 (15,9-16,2)	11,7 (11,5-12,1)	16,2 (15,9-16,3)	11,7 (11,6-12,1)	16,3 (16,0-16,4)	11,7 (11,6-12,1)	16,4 (16,2-16,5)	11,8 (11,6-12,0)	16,6 (16,4-16,7)	11,7 (11,5-11,9)	16,1 (15,9-16,2)	11,7 (11,4-12,0)	16,3 (15,9-16,5)	11,6 (11,3-11,9)	16,4 (16,0-16,8)	11,6 (11,1-12,0)	16,7 (16,2-17,0)	11,3 (10,8-11,7)	17,5 (16,9-18,0)
	30%	11,4 (11,2-11,6)	16,0 (15,9-16,1)	11,3 (11,1-11,5)	16,1 (16,0-16,2)	11,3 (11,1-11,5)	16,3 (16,1-16,3)	11,3 (11,1-11,5)	16,4 (16,3-16,5)	11,3 (11,1-11,5)	16,5 (16,4-16,6)	11,4 (11,1-11,6)	16,7 (16,6-16,8)	11,2 (11,0-11,5)	16,2 (16,0-16,3)	11,2 (10,9-11,5)	16,4 (16,2-16,6)	11,0 (10,6-11,4)	16,7 (16,2-17,0)	10,9 (10,3-11,3)	17,1 (16,5-17,4)	10,2 (9,9-11,0)	18,1 (17,3-18,5)
9.0-13.0	5%	12,1 (11,9-12,3)	15,9 (15,6-16,0)	12,1 (11,9-12,4)	16,0 (15,6-16,1)	12,1 (11,9-12,4)	16,1 (15,7-16,2)	12,1 (11,9-12,4)	16,2 (15,8-16,3)	12,2 (12,0-12,4)	16,2 (15,9-16,4)	12,2 (12,0-12,5)	16,4 (16,0-16,6)	12,0 (11,9-12,4)	16,0 (15,7-16,2)	12,1 (11,9-12,4)	16,1 (15,8-16,4)	12,1 (11,9-12,4)	16,2 (15,9-16,6)	12,0 (11,9-12,3)	16,4 (16,1-16,7)	12,0 (11,9-12,2)	16,9 (16,6-17,3)
	10%	12,0 (11,9-12,3)	15,9 (15,6-16,0)	12,0 (11,9-12,3)	16,0 (15,6-16,1)	12,0 (11,9-12,4)	16,1 (15,8-16,2)	12,1 (11,9-12,4)	16,2 (15,8-16,3)	12,1 (11,9-12,4)	16,3 (16,0-16,4)	12,2 (11,9-12,4)	16,5 (16,2-16,6)	12,0 (11,9-12,3)	16,0 (15,7-16,2)	12,0 (11,8-12,3)	16,1 (15,8-16,4)	12,0 (11,8-12,3)	16,3 (15,9-16,6)	12,0 (11,8-12,3)	16,4 (16,1-16,8)	11,9 (11,8-12,2)	17,0 (16,6-17,4)
	15%	12,0 (11,8-12,2)	15,9 (15,6-16,0)	12,0 (11,8-12,2)	16,0 (15,8-16,1)	12,0 (11,8-12,3)	16,1 (15,8-16,2)	12,0 (11,8-12,3)	16,2 (15,9-16,4)	12,1 (11,9-12,3)	16,3 (16,0-16,4)	12,1 (11,9-12,3)	16,5 (16,2-16,7)	12,0 (11,8-12,2)	16,1 (15,7-16,2)	12,0 (11,8-12,3)	16,2 (15,8-16,4)	12,0 (11,8-12,2)	16,3 (15,9-16,7)	11,9 (11,7-12,2)	16,4 (16,1-16,9)	11,9 (11,7-12,1)	17,1 (16,7-17,6)
	20%	11,9 (11,8-12,1)	15,9 (15,7-16,0)	11,9 (11,8-12,1)	16,0 (15,8-16,2)	11,9 (11,8-12,1)	16,1 (15,9-16,3)	12,0 (11,8-12,2)	16,3 (16,0-16,4)	12,0 (11,8-12,2)	16,4 (16,1-16,5)	12,0 (11,9-12,3)	16,6 (16,3-16,7)	11,9 (11,8-12,1)	16,1 (15,9-16,2)	11,9 (11,7-12,1)	16,2 (15,9-16,5)	11,9 (11,6-12,2)	16,3 (16,0-16,7)	11,9 (11,6-12,2)	16,5 (16,1-17,0)	11,8 (11,6-12,0)	17,3 (16,8-17,9)
	30%	11,6 (11,5-11,8)	16,0 (16,0-16,1)	11,6 (11,5-11,7)	16,2 (16,1-16,3)	11,6 (11,5-11,7)	16,3 (16,2-16,4)	11,7 (11,5-11,8)	16,4 (16,3-16,5)	11,7 (11,6-11,8)	16,5 (16,4-16,6)	11,8 (11,6-11,9)	16,7 (16,7-16,9)	11,6 (11,4-11,7)	16,2 (16,1-16,3)	11,5 (11,4-11,6)	16,4 (16,3-16,5)	11,4 (11,3-11,6)	16,7 (16,5-16,9)	11,4 (11,2-11,6)	17,0 (16,6-17,4)	11,2 (11,0-11,6)	17,9 (17,2-18,2)
8.0-12.0	5%	12,1 (11,9-12,3)	15,9 (15,6-16,0)	12,1 (12,0-12,4)	16,0 (15,6-16,1)	12,1 (11,9-12,4)	16,1 (15,7-16,2)	12,1 (11,9-12,4)	16,2 (15,8-16,3)	12,2 (12,0-12,4)	16,3 (15,9-16,4)	12,2 (12,0-12,5)	16,4 (16,0-16,6)	12,1 (11,9-12,4)	16,0 (15,7-16,2)	12,1 (11,9-12,3)	16,1 (15,8-16,4)	12,1 (11,9-12,4)	16,2 (15,9-16,6)	12,0 (11,9-12,3)	16,4 (16,1-16,7)	12,0 (12,0-12,1)	16,9 (16,6-17,2)
	10%	12,0 (11,9-12,3)	15,9 (15,6-16,0)	12,1 (11,9-12,3)	16,0 (15,6-16,1)	12,1 (11,9-12,3)	16,1 (15,8-16,2)	12,1 (11,9-12,4)	16,2 (15,8-16,3)	12,1 (12,0-12,4)	16,3 (15,9-16,4)	12,2 (12,0-12,4)	16,5 (16,1-16,6)	12,0 (11,9-12,3)	16,0 (15,7-16,2)	12,1 (11,9-12,3)	16,1 (15,8-16,4)	12,0 (11,9-12,3)	16,2 (15,9-16,6)	12,0 (11,9-12,3)	16,4 (16,1-16,8)	12,0 (11,9-12,2)	16,9 (16,6-17,2)
	15%	12,0 (11,9-12,2)	15,9 (15,6-16,0)	12,1 (11,9-12,3)	16,0 (15,7-16,2)	12,1 (11,9-12,3)	16,1 (15,8-16,3)	12,1 (11,9-12,3)	16,2 (15,9-16,4)	12,1 (12,0-12,3)	16,3 (16,0-16,5)	12,2 (12,0-12,4)	16,5 (16,1-16,6)	12,1 (11,9-12,3)	16,0 (15,8-16,2)	12,0 (11,9-12,3)	16,1 (15,8-16,4)	12,0 (11,9-12,2)	16,3 (15,9-16,6)	12,0 (11,9-12,2)	16,4 (16,1-16,8)	12,0 (11,9-12,1)	17,0 (16,6-17,4)
	20%	12,0 (11,9-12,1)	15,9 (15,6-16,0)	12,0 (11,9-12,2)	16,0 (15,8-16,2)	12,0 (11,9-12,2)	16,1 (15,9-16,3)	12,1 (11,9-12,2)	16,2 (15,9-16,4)	12,1 (12,0-12,2)	16,3 (16,1-16,5)	12,1 (12,0-12,3)	16,5 (16,2-16,7)	12,0 (11,9-12,2)	16,1 (15,7-16,2)	12,0 (11,9-12,2)	16,2 (15,9-16,5)	12,0 (11,8-12,2)	16,3 (15,9-16,6)	12,0 (11,9-12,2)	16,5 (16,1-16,9)	12,0 (11,8-12,1)	17,1 (16,7-17,6)
	30%	11,8 (11,8-11,9)	16,0 (15,9-16,1)	11,8 (11,7-11,9)	16,1 (16,1-16,2)	11,9 (11,8-11,9)	16,2 (16,2-16,3)	11,9 (11,8-11,9)	16,3 (16,3-16,4)	11,9 (11,9-12,0)	16,5 (16,4-16,5)	12,0 (11,9-12,1)	16,7 (16,6-16,8)	11,8 (11,7-11,9)	16,2 (16,1-16,3)	11,8 (11,7-11,9)	16,3 (16,2-16,5)	11,8 (11,7-11,9)	16,4 (16,3-16,7)	11,8 (11,7-11,9)	16,6 (16,4-16,9)	11,8 (11,7-12,0)	17,2 (16,7-17,9)

Abnormal,%		15.0-19.0										16.0-20.0									
		5%		10%		15%		20%		30%		5%		10%		15%		20%		30%	
None	0%	12,1 (12,0-12,4)	15,9 (15,6-16,2)	12,1 (12,0-12,4)	16,0 (15,7-16,2)	12,1 (12,0-12,4)	16,0 (15,8-16,3)	12,1 (12,0-12,4)	16,1 (15,9-16,3)	12,0 (12,0-12,1)	16,5 (16,3-16,6)	12,1 (12,0-12,4)	15,9 (15,6-16,1)	12,1 (12,0-12,4)	16,0 (15,7-16,1)	12,1 (12,0-12,4)	16,0 (15,8-16,1)	12,1 (12,0-12,4)	16,0 (15,8-16,1)	12,1 (12,0-12,3)	16,2 (16,1-16,3)
11.0-15.0	5%	12,1 (11,9-12,3)	16,0 (15,6-16,1)	12,1 (11,9-12,4)	16,0 (15,7-16,2)	12,1 (11,9-12,3)	16,1 (15,8-16,3)	12,0 (11,9-12,3)	16,1 (15,8-16,3)	11,9 (11,9-12,0)	16,5 (16,3-16,6)	12,1 (11,9-12,3)	15,9 (15,6-16,0)	12,1 (11,9-12,3)	16,0 (15,7-16,1)	12,1 (11,9-12,3)	16,0 (15,7-16,1)	12,0 (11,9-12,4)	16,0 (15,8-16,1)	12,0 (11,9-12,1)	16,2 (16,1-16,3)
	10%	11,9 (11,8-12,3)	15,9 (15,6-16,1)	11,9 (11,8-12,3)	16,0 (15,7-16,2)	12,0 (11,8-12,3)	16,0 (15,8-16,3)	11,9 (11,7-12,2)	16,1 (15,8-16,4)	11,8 (11,8-11,9)	16,5 (16,4-16,6)	11,9 (11,8-12,3)	15,9 (15,6-16,1)	12,0 (11,8-12,3)	15,9 (15,7-16,1)	12,0 (11,8-12,2)	16,0 (15,7-16,1)	12,0 (11,8-12,2)	16,0 (15,8-16,2)	11,9 (11,7-12,1)	16,1 (16,0-16,2)
	15%	11,9 (11,7-12,2)	15,9 (15,7-16,1)	11,9 (11,7-12,2)	16,0 (15,7-16,2)	11,9 (11,7-12,2)	16,0 (15,7-16,3)	11,8 (11,7-12,2)	16,1 (15,8-16,4)	11,7 (11,6-11,8)	16,5 (16,3-16,6)	11,9 (11,7-12,2)	15,9 (15,7-16,0)	11,9 (11,7-12,2)	15,9 (15,7-16,0)	11,8 (11,7-12,2)	16,0 (15,7-16,1)	11,9 (11,6-12,2)	16,0 (15,8-16,1)	11,8 (11,7-12,1)	16,1 (16,0-16,2)
	20%	11,8 (11,6-12,2)	15,9 (15,6-16,1)	11,8 (11,6-12,2)	16,0 (15,6-16,2)	11,8 (11,6-12,1)	16,0 (15,7-16,3)	11,7 (11,5-12,0)	16,1 (15,8-16,4)	11,6 (11,5-11,7)	16,5 (16,3-16,6)	11,8 (11,6-12,2)	15,9 (15,5-16,0)	11,8 (11,6-12,2)	15,9 (15,6-16,0)	11,8 (11,6-12,1)	15,9 (15,7-16,1)	11,7 (11,6-12,1)	15,9 (15,7-16,1)	11,7 (11,5-11,9)	16,1 (16,0-16,2)
	30%	11,6 (11,4-12,0)	15,9 (15,6-16,0)	11,6 (11,4-12,0)	15,9 (15,6-16,2)	11,6 (11,4-11,9)	16,0 (15,7-16,2)	11,5 (11,4-11,8)	16,1 (15,8-16,3)	11,4 (11,3-11,5)	16,3 (16,1-16,5)	11,6 (11,4-12,0)	15,8 (15,6-16,0)	11,6 (11,4-12,0)	15,9 (15,6-16,0)	11,5 (11,4-11,9)	15,9 (15,7-16,0)	11,5 (11,4-11,9)	15,9 (15,7-16,0)	11,5 (11,3-11,7)	16,0 (15,9-16,1)
10.0-14.0	5%	12,0 (11,8-12,3)	16,0 (15,6-16,1)	12,0 (11,8-12,4)	16,0 (15,7-16,2)	12,0 (11,8-12,3)	16,1 (15,8-16,3)	12,0 (11,8-12,3)	16,1 (15,8-16,4)	11,9 (11,8-12,0)	16,5 (16,4-16,7)	12,1 (11,9-12,3)	15,9 (15,7-16,0)	12,1 (11,8-12,3)	15,9 (15,7-16,1)	12,1 (11,8-12,4)	16,0 (15,8-16,1)	12,1 (11,8-12,4)	16,0 (15,8-16,2)	12,0 (11,8-12,1)	16,2 (16,1-16,3)
	10%	12,0 (11,8-12,3)	16,0 (15,7-16,1)	11,9 (11,7-12,3)	16,0 (15,8-16,2)	11,9 (11,7-12,3)	16,1 (15,8-16,3)	11,9 (11,7-12,2)	16,2 (15,9-16,4)	11,8 (11,5-11,9)	16,6 (16,4-16,8)	12,0 (11,8-12,3)	15,9 (15,7-16,0)	12,0 (11,8-12,3)	16,0 (15,8-16,1)	12,0 (11,7-12,3)	16,0 (15,8-16,1)	11,9 (11,7-12,3)	16,0 (15,8-16,2)	11,9 (11,6-12,1)	16,2 (16,1-16,3)
	15%	11,9 (11,6-12,1)	16,0 (15,8-16,1)	11,8 (11,6-12,2)	16,1 (15,8-16,3)	11,8 (11,5-12,1)	16,1 (15,9-16,3)	11,8 (11,5-12,1)	16,2 (15,9-16,4)	11,6 (11,3-11,9)	16,6 (16,4-16,9)	11,8 (11,7-12,1)	16,0 (15,8-16,1)	11,8 (11,6-12,2)	16,0 (15,8-16,1)	11,8 (11,6-12,2)	16,0 (15,8-16,2)	11,9 (11,6-12,1)	16,0 (15,8-16,2)	11,9 (11,5-12,1)	16,2 (16,1-16,3)
	20%	11,7 (11,5-12,0)	16,0 (15,8-16,1)	11,7 (11,5-12,1)	16,1 (15,8-16,2)	11,7 (11,4-12,0)	16,2 (15,9-16,4)	11,6 (11,3-12,0)	16,2 (15,9-16,5)	11,4 (11,0-11,7)	16,7 (16,4-17,0)	11,7 (11,5-12,0)	16,0 (15,8-16,1)	11,7 (11,5-12,0)	16,0 (15,8-16,1)	11,7 (11,5-12,0)	16,0 (15,9-16,1)	11,7 (11,4-12,0)	16,0 (15,8-16,2)	11,7 (11,3-11,9)	16,2 (16,1-16,3)
	30%	11,3 (11,0-11,5)	16,1 (16,0-16,2)	11,2 (11,0-11,5)	16,2 (16,0-16,3)	11,1 (10,8-11,5)	16,3 (16,1-16,5)	11,0 (10,6-11,4)	16,4 (16,1-16,7)	10,7 (10,1-11,1)	16,9 (16,5-17,4)	11,3 (11,1-11,5)	16,0 (15,9-16,1)	11,3 (11,1-11,5)	16,1 (16,0-16,1)	11,2 (11,1-11,5)	16,1 (16,0-16,2)	11,2 (10,9-11,4)	16,1 (16,0-16,2)	11,1 (10,6-11,5)	16,2 (16,1-16,3)
9.0-13.0	5%	12,0 (11,9-12,3)	16,0 (15,6-16,1)	12,1 (11,9-12,4)	16,0 (15,7-16,2)	12,1 (11,9-12,3)	16,1 (15,8-16,3)	12,0 (11,9-12,4)	16,1 (15,9-16,4)	12,0 (11,9-12,1)	16,5 (16,4-16,7)	12,1 (11,9-12,3)	15,9 (15,6-16,1)	12,1 (11,9-12,4)	16,0 (15,7-16,1)	12,1 (11,9-12,4)	16,0 (15,8-16,1)	12,1 (11,9-12,4)	16,0 (15,8-16,2)	12,0 (11,9-12,2)	16,2 (16,1-16,3)
	10%	12,0 (11,9-12,3)	16,0 (15,7-16,1)	12,0 (11,8-12,3)	16,0 (15,7-16,2)	12,0 (11,8-12,3)	16,1 (15,8-16,3)	12,0 (11,8-12,3)	16,2 (15,9-16,4)	11,9 (11,8-12,1)	16,6 (16,4-16,7)	12,0 (11,9-12,3)	15,9 (15,7-16,1)	12,0 (11,9-12,3)	16,0 (15,7-16,1)	12,0 (11,8-12,3)	16,0 (15,8-16,1)	12,0 (11,8-12,3)	16,0 (15,8-16,2)	12,0 (11,8-12,2)	16,2 (16,1-16,3)
	15%	12,0 (11,8-12,2)	16,0 (15,7-16,2)	12,0 (11,8-12,3)	16,0 (15,8-16,3)	12,0 (11,7-12,2)	16,1 (15,8-16,3)	12,0 (11,7-12,3)	16,2 (15,9-16,4)	11,9 (11,7-12,0)	16,6 (16,4-16,8)	12,0 (11,8-12,2)	16,0 (15,8-16,1)	12,0 (11,8-12,3)	16,0 (15,8-16,1)	12,0 (11,8-12,2)	16,0 (15,8-16,1)	12,0 (11,8-12,3)	16,0 (15,8-16,2)	12,0 (11,8-12,2)	16,2 (16,1-16,3)
	20%	11,9 (11,8-12,1)	16,0 (15,8-16,2)	11,9 (11,7-12,1)	16,1 (15,8-16,2)	11,9 (11,7-12,1)	16,1 (15,8-16,4)	11,9 (11,7-12,1)	16,2 (15,9-16,5)	11,8 (11,6-12,0)	16,7 (16,4-16,9)	11,9 (11,8-12,1)	16,0 (15,8-16,1)	11,9 (11,8-12,1)	16,0 (15,8-16,1)	11,9 (11,8-12,2)	16,0 (15,8-16,2)	11,9 (11,7-12,1)	16,0 (15,8-16,2)	11,9 (11,7-12,1)	16,2 (16,1-16,3)
	30%	11,6 (11,4-11,7)	16,1 (16,1-16,2)	11,5 (11,4-11,6)	16,3 (16,2-16,4)	11,5 (11,4-11,6)	16,4 (16,2-16,5)	11,4 (11,3-11,6)	16,4 (16,3-16,6)	11,3 (11,1-11,6)	16,8 (16,5-17,2)	11,6 (11,5-11,7)	16,1 (16,0-16,2)	11,6 (11,5-11,7)	16,1 (16,1-16,2)	11,5 (11,4-11,6)	16,2 (16,1-16,2)	11,5 (11,4-11,6)	16,2 (16,1-16,3)	11,4 (11,2-11,6)	16,3 (16,1-16,4)
8.0-12.0	5%	12,1 (11,9-12,4)	15,9 (15,6-16,1)	12,1 (11,9-12,3)	16,0 (15,8-16,2)	12,1 (11,9-12,3)	16,1 (15,8-16,3)	12,1 (11,9-12,4)	16,1 (15,8-16,4)	12,0 (11,9-12,1)	16,5 (16,4-16,7)	12,1 (11,9-12,4)	15,9 (15,6-16,1)	12,1 (11,9-12,4)	16,0 (15,7-16,1)	12,1 (11,9-12,3)	16,0 (15,8-16,1)	12,1 (11,9-12,4)	16,0 (15,8-16,2)	12,1 (11,9-12,2)	16,2 (16,1-16,3)
	10%	12,0 (11,9-12,3)	16,0 (15,7-16,1)	12,1 (11,9-12,3)	16,0 (15,7-16,2)	12,1 (11,9-12,3)	16,1 (15,8-16,3)	12,0 (11,9-12,3)	16,2 (15,9-16,4)	12,0 (11,9-12,1)	16,5 (16,4-16,7)	12,1 (11,9-12,3)	16,0 (15,7-16,1)	12,0 (11,9-12,3)	16,0 (15,8-16,1)	12,1 (11,9-12,3)	16,0 (15,8-16,1)	12,1 (11,9-12,3)	16,0 (15,8-16,2)	12,1 (11,9-12,2)	16,2 (16,1-16,3)
	15%	12,0 (11,9-12,3)	16,0 (15,8-16,2)	12,0 (11,9-12,3)	16,0 (15,8-16,2)	12,0 (11,9-12,3)	16,1 (15,8-16,3)	12,0 (11,9-12,2)	16,2 (15,9-16,4)	12,0 (11,8-12,1)	16,6 (16,4-16,8)	12,1 (11,9-12,3)	16,0 (15,8-16,1)	12,0 (11,9-12,3)	16,0 (15,7-16,1)	12,0 (11,9-12,3)	16,0 (15,8-16,1)	12,0 (11,9-12,2)	16,0 (15,8-16,2)	12,1 (11,9-12,2)	16,2 (16,1-16,3)
	20%	12,0 (11,9-12,2)	16,0 (15,7-16,1)	12,0 (11,8-12,2)	16,1 (15,8-16,2)	12,0 (11,8-12,2)	16,1 (15,8-16,3)	12,0 (11,9-12,3)	16,2 (15,9-16,5)	12,0 (11,8-12,1)	16,6 (16,4-16,8)	12,0 (11,9-12,1)	16,0 (15,7-16,1)	12,0 (11,9-12,2)	16,0 (15,8-16,1)	12,0 (11,8-12,2)	16,0 (15,8-16,2)	12,0 (11,9-12,2)	16,0 (15,8-16,2)	12,0 (11,9-12,2)	16,2 (16,1-16,3)
	30%	11,8 (11,7-11,9)	16,1 (16,0-16,2)	11,8 (11,7-11,9)	16,2 (16,0-16,3)	11,8 (11,7-11,9)	16,2 (16,1-16,3)	11,8 (11,7-11,9)	16,3 (16,1-16,4)	11,8 (11,6-11,9)	16,6 (16,3-17,0)	11,8 (11,7-11,9)	16,0 (16,0-16,1)	11,8 (11,7-11,9)	16,1 (16,0-16,1)	11,8 (11,7-11,9)	16,1 (16,0-16,2)	11,8 (11,7-11,8)	16,1 (16,0-16,2)	11,8 (11,6-11,9)	16,2 (16,1-16,3)

Legend	Green (11.8-12.2 and 15.8-16.2)	74%	Appropriate for RI estimation.	Yellow (11.6-12.4 and 15.6-16.4)	13%	Appropriate for QC control.	Red (<11.6 or >12.4 and <15.6 or >16.4)	13%	Inappropriate RIs. Use with caution in select settings.

Supplemental Table 1: Reference intervals in simulated "Hemoglobin" datasets. Random "physiological" test results (true reference interval 12.0-16.0 "g/dL") and two populations of "abnormal" test results (95 % interval of "abnormal" test results: first column and second row) were mixed (proportion of "abnormal" test results: second column and third row), and reference intervals of the combined population (n=10,000) were estimated using *kosmic*. Colored columns denote the median reference interval, ranges below denote the 90% confidence interval of calculated reference intervals.

TSH, 0.25-4.0 µU/ml, n=50,000												
**Abnormal distribution**	**Abnormal, %**											
	**0%**		**5%**		**10%**		**15%**		**20%**		**30%**	
**2.0-4.0**	0,25	3,79	0,25	3,85	0,25	3,93	0,25	4,11	0,24	4,53	0,16	9,65
	0,23-0,27	3,25-3,99	0,24-0,27	3,38-4,18	0,23-0,26	3,50-4,19	0,24-0,26	3,83-4,30	0,23-0,25	4,31-4,82	0,14-0,20	9,01-11,20
**1.0-5.0**			0,25	3,94	0,25	4,21	0,24	4,52	0,24	5,06	0,22	8,48
			0,24-0,27	3,48-4,30	0,24-0,26	3,69-4,45	0,24-0,25	4,22-4,79	0,24-0,25	4,72-5,36	0,22-0,23	7,57-9,14
**0.0-6.0**			0,25	3,95	0,25	4,13	0,25	4,42	0,24	4,75	0,24	6,09
			0,23-0,27	3,40-4,30	0,24-0,26	3,75-4,45	0,24-0,25	4,00-4,65	0,24-0,25	4,37-5,01	0,23-0,24	5,74-6,48
**-1.0-7.0**			0,25	3,90	0,25	4,08	0,25	4,25	0,24	4,51	0,24	5,33
			0,23-0,27	3,39-4,26	0,24-0,27	3,59-4,39	0,24-0,26	3,80-4,56	0,24-0,26	4,19-4,82	0,23-0,25	5,05-5,65
**2.5-4.5**			0,25	3,82	0,25	3,86	0,25	3,94	0,25	4,04	0,20	8,33
			0,23-0,27	3,36-4,12	0,23-0,26	3,42-4,13	0,24-0,26	3,50-4,11	0,24-0,26	3,80-4,21	0,19-0,23	6,31-9,28
**1.5-5.5**			0,25	3,87	0,25	4,05	0,25	4,26	0,24	4,61	0,22	7,65
			0,24-0,27	3,37-4,22	0,24-0,27	3,52-4,32	0,24-0,25	3,98-4,47	0,24-0,25	4,39-4,89	0,21-0,22	6,94-8,12
**0.5-6.5**			0,25	3,91	0,25	4,08	0,25	4,32	0,24	4,62	0,23	6,13
			0,23-0,27	3,30-4,25	0,24-0,27	3,70-4,37	0,24-0,26	3,80-4,54	0,24-0,25	4,38-4,85	0,23-0,24	5,61-6,42
**-0.5-7.5**			0,25	3,91	0,25	4,04	0,25	4,26	0,25	4,50	0,24	5,38
			0,23-0,27	3,37-4,22	0,24-0,27	3,64-4,35	0,24-0,26	3,79-4,51	0,24-0,25	4,21-4,76	0,23-0,24	5,12-5,67
**3.0-5.0**			0,25	3,82	0,25	3,85	0,25	3,91	0,25	3,92	0,24	5,26
			0,23-0,27	3,38-4,09	0,23-0,27	3,43-4,11	0,23-0,27	3,47-4,11	0,24-0,26	3,65-4,13	0,21-0,25	5,03-6,30
**2.0-6.0**			0,25	3,86	0,25	3,95	0,25	4,07	0,24	4,27	0,21	6,59
			0,23-0,27	3,32-4,15	0,24-0,26	3,53-4,20	0,24-0,26	3,75-4,28	0,24-0,25	4,08-4,44	0,21-0,23	6,02-6,94
**1.0-7.0**			0,25	3,90	0,25	4,04	0,25	4,22	0,24	4,45	0,23	5,98
			0,24-0,27	3,44-4,18	0,24-0,27	3,60-4,28	0,24-0,26	3,93-4,41	0,24-0,25	4,23-4,66	0,22-0,24	5,61-6,27
**0.0-8.0**			0,25	3,89	0,25	4,06	0,25	4,21	0,25	4,42	0,24	5,38
			0,23-0,27	3,41-4,20	0,24-0,27	3,49-4,35	0,24-0,26	3,66-4,42	0,24-0,25	4,19-4,64	0,23-0,24	5,06-5,64
**3.5-5.5**			0,25	3,81	0,25	3,89	0,25	3,92	0,25	3,93	0,24	4,77
			0,23-0,27	3,38-4,07	0,23-0,27	3,43-4,06	0,23-0,27	3,51-4,08	0,23-0,26	3,64-4,10	0,24-0,25	4,65-4,99
**2.5-6.5**			0,25	3,84	0,25	3,91	0,25	3,96	0,25	4,06	0,22	5,76
			0,23-0,27	3,33-4,12	0,23-0,27	3,45-4,13	0,24-0,26	3,58-4,15	0,24-0,26	3,86-4,24	0,21-0,24	5,08-6,07
**1.5-7.5**			0,25	3,84	0,25	3,96	0,25	4,10	0,24	4,32	0,23	5,68
			0,23-0,27	3,45-4,17	0,24-0,27	3,56-4,20	0,24-0,26	3,73-4,30	0,24-0,25	4,10-4,50	0,22-0,24	5,30-5,93
**0.5-8.5**			0,25	3,89	0,25	3,98	0,25	4,14	0,25	4,35	0,23	5,35
			0,24-0,27	3,37-4,17	0,23-0,27	3,54-4,24	0,24-0,26	3,77-4,36	0,24-0,25	4,14-4,56	0,23-0,24	4,98-5,56
**4.0-6.0**			0,25	3,83	0,25	3,87	0,25	3,92	0,25	3,93	0,24	4,53
			0,23-0,27	3,36-4,04	0,23-0,27	3,39-4,04	0,23-0,27	3,51-4,06	0,24-0,26	3,65-4,09	0,24-0,25	4,44-4,64
**3.0-7.0**			0,25	3,82	0,25	3,89	0,25	3,94	0,25	3,95	0,23	5,13
			0,23-0,27	3,34-4,09	0,23-0,27	3,42-4,10	0,24-0,27	3,50-4,13	0,24-0,26	3,69-4,15	0,22-0,25	4,71-5,39
**2.0-8.0**			0,25	3,86	0,25	3,95	0,25	4,00	0,25	4,16	0,22	5,37
			0,23-0,27	3,32-4,15	0,23-0,27	3,49-4,19	0,24-0,26	3,63-4,23	0,24-0,26	3,94-4,34	0,22-0,24	5,06-5,60
**1.0-9.0**			0,25	3,87	0,25	3,97	0,25	4,10	0,25	4,25	0,23	5,21
			0,23-0,27	3,35-4,15	0,24-0,26	3,51-4,25	0,24-0,26	3,72-4,28	0,24-0,25	4,02-4,47	0,23-0,24	4,92-5,43
**4.5-6.5**			0,25	3,83	0,25	3,88	0,25	3,93	0,25	3,93	0,24	4,37
			0,23-0,27	3,36-4,03	0,23-0,27	3,41-4,04	0,23-0,27	3,51-4,05	0,24-0,26	3,66-4,06	0,24-0,25	4,28-4,46
**3.5-7.5**			0,25	3,82	0,25	3,86	0,25	3,93	0,25	3,94	0,24	4,65
			0,23-0,27	3,36-4,06	0,23-0,27	3,39-4,06	0,24-0,27	3,53-4,10	0,24-0,26	3,67-4,13	0,22-0,25	4,50-4,94
**2.5-8.5**			0,25	3,85	0,25	3,92	0,25	3,98	0,25	4,06	0,23	5,07
			0,23-0,27	3,44-4,11	0,23-0,27	3,47-4,14	0,24-0,27	3,60-4,16	0,24-0,26	3,80-4,24	0,22-0,24	4,74-5,29
**1.5-9.5**			0,25	3,85	0,25	3,96	0,25	4,06	0,25	4,17	0,23	5,07
			0,23-0,27	3,34-4,14	0,24-0,27	3,48-4,18	0,24-0,27	3,68-4,27	0,24-0,26	3,95-4,35	0,23-0,24	4,83-5,27
**5.0-7.0**			0,25	3,84	0,25	3,87	0,25	3,93	0,25	3,94	0,25	4,26
			0,23-0,27	3,36-4,03	0,23-0,27	3,39-4,04	0,23-0,27	3,51-4,04	0,23-0,26	3,67-4,06	0,24-0,25	4,19-4,34
**4.0-8.0**			0,25	3,83	0,25	3,87	0,25	3,93	0,25	3,93	0,24	4,45
			0,23-0,27	3,36-4,05	0,23-0,27	3,40-4,05	0,23-0,27	3,52-4,06	0,24-0,26	3,64-4,11	0,23-0,25	4,36-4,64
**3.0-9.0**			0,25	3,82	0,25	3,90	0,25	3,95	0,25	4,00	0,23	4,80
			0,23-0,27	3,40-4,10	0,23-0,27	3,39-4,12	0,24-0,27	3,48-4,13	0,24-0,26	3,75-4,17	0,22-0,25	4,52-4,98
**2.0-10.0**			0,25	3,85	0,25	3,91	0,25	4,00	0,25	4,09	0,23	4,91
			0,23-0,27	3,32-4,12	0,23-0,27	3,51-4,18	0,24-0,27	3,58-4,21	0,24-0,26	3,88-4,28	0,23-0,24	4,64-5,08

**Legend**			
	**Green (3.80-4.20 and 0.20-0.30)**	81,9%	Appropriate for RI estimation.
	**Yellow (3.60-4.40 and 0.15-0.35)**	5,3%	Appropriate for QC control.
	**Red (<3.60 or >4.40 and <0.15 or >0.35)**	12,8%	Inappropriate RIs. Use with caution in select settings.

Supplemental Table 2: Reference intervals in simulated "Thyroid-stimulating hormone, TSH" datasets. Random "physiological" test results (true reference interval 0.25-4.0 "µU/mL") and populations of "abnormal" test results (95 % interval of "abnormal" test results: first column) were mixed (proportion of "pathological" test results: third row), and reference intervals of the combined population (n=50,000) were estimated using *kosmic*. Colored columns denote the median reference interval, ranges below denote the 90% confidence interval of calculated reference intervals.

y-GT, 10-50 U/l , n=25,000												
**Abnormal distribution**	**Abnormal, %**											
	**0%**		**5%**		**10%**		**15%**		**20%**		**30%**	
**1-5**	10	47	9	47	9	47	9	47	9	47	9	42
	9-11	41-49	9-10	41-49	9-10	42-50	9-10	40-50	9-10	37-50	9-9	36-49
**3-13**			9	47	9	46	9	44	8	44	4	39
			8-10	39-49	8-10	39-49	7-10	38-49	5-10	36-49	1-6	37-42
**5-25**			9	47	8	46	7	45	6	45	5	45
			8-10	39-49	7-10	38-49	6-9	38-49	5-8	39-49	3-7	37-48
**15-75**			10	49	10	50	10	52	10	54	10	57
			9-10	41-51	9-10	42-53	9-10	44-55	9-11	44-57	9-11	50-60
**20-100**			10	49	10	51	10	53	10	55	10	63
			9-10	43-52	9-10	44-53	9-10	46-56	9-10	51-58	9-10	60-67
**25-125**			10	49	10	50	10	51	10	53	9	61
			9-10	42-51	9-10	43-52	9-10	46-54	9-10	49-57	9-10	59-64
**30-150**			10	48	10	49	10	50	10	52	9	58
			9-10	42-51	9-10	43-52	9-10	45-52	9-10	49-54	9-10	56-60
**50-250**			10	48	10	48	10	49	10	49	9	52
			9-10	41-50	9-10	43-50	9-10	44-50	9-10	46-50	9-10	51-53

**Legend**			
	**Green (8-12 and 48-52)**	65,9%	Appropriate for RI estimation.
	**Yellow (6-14 and 46-54)**	17,1%	Appropriate for QC control.
	**Red (<6 or >14 and <46 or >54)**	17,1%	Inappropriate RIs. Use with caution in select settings.

Supplemental Table 3: Reference intervals in simulated "Gamma-glutamyltransferase, y-GT" datasets. Random "physiological" test results (true reference interval 10-50 "U/L") and populations of "abnormal" test results (95 % interval of "abnormal" test results: first column) were mixed (proportion of "abnormal" test results: third row), and reference intervals of the combined population (n=25,000) were estimated using *kosmic*. Colored columns denote the median reference interval, ranges below denote the 90% confidence interval of calculated reference intervals.
